# Improving PrEP access for adolescent girls and young women: a descriptive analysis of community‐based PrEP delivery in the DREAMS programme in Zambia

**DOI:** 10.1002/jia2.26484

**Published:** 2025-07-07

**Authors:** Maurice Musheke, Jake M. Pry, Izukanji Sikazwe, Walusiku J. Muyunda, Kanema Chiyenu, Charity M. Siame, Winfred K. Khondowe, Bwalya Mushiki, Martha M. Mwaba, Pelile Zulu, Flavia Mwape, Bridget Siamasuku, Davies Shula, Mable B. Mweemba, Cuthbert Kanene, Arlene Phiri, Michael E. Herce

**Affiliations:** ^1^ Centre for Infectious Disease Research in Zambia (CIDRZ) Lusaka Zambia; ^2^ School of Medicine University of California Davis California USA; ^3^ Young Women Christian Association Lusaka Zambia; ^4^ Ministry of Health, Ndeke House Lusaka Zambia; ^5^ United States Agency for International Development United States of America Embassy Lusaka Zambia; ^6^ Institute for Global Health and Infectious Diseases University of North Carolina Chapel Hill North Carolina USA

**Keywords:** adolescent girls and young women, community centre, HIV prevention, pre‐exposure prophylaxis, adherence, Zambia

## Abstract

**Introduction:**

Despite being at high risk of HIV acquisition, access to pre‐exposure prophylaxis (PrEP) among adolescent girls and young women (AGYW) is low in Zambia because PrEP is traditionally delivered in clinical settings. We describe the effects of community centres supported by the Determined, Resilient, Empowered, AIDS‐free, Mentored, and Safe (DREAMS) initiative on PrEP outcomes in Zambia and examine factors associated with PrEP continuation.

**Methods:**

We collected individual‐level PrEP data for AGYW aged 15–24 years at risk of HIV acquisition and enrolled in DREAMS in seven districts of Zambia between August 2022 and August 2024. We used Pearson's Chi‐squared test to examine differences in beneficiary characteristics between clients with a PrEP initiation visit and ≥ 2 PrEP visits (i.e. an initiation plus ≥ 1 return visit), and mixed effects Poisson regression modelling to estimate the association between DREAMS enrolment criteria and PrEP continuation (defined as ≥ 1 PrEP visit within 180 days of initiation). We also estimated the marginal probability of PrEP continuation by number of DREAMS enrolment criteria and used Kaplan‐Meier methods to estimate the time to the first PrEP return visit by client age band.

**Results:**

Between 11 August 2022 and 23 August 2024, 15,502 AGYW aged 15–24 years were screened for PrEP eligibility, of whom 15,072 (97.2%) initiated PrEP per national guidelines. Of those initiating PrEP, 9807 (65.1%) had sufficient follow‐up time to allow for observation of a PrEP return visit. The proportion of AGYW who had ≥ 1 PrEP return visit within 180 days of initiation was 59.0% (*n*/*N* = 5706/9675). Across age bands, the percent probability of having a PrEP return visit within 180 days of initiation was highest among clients who reported ≥ 4 DREAMS enrolment criteria at 91.7% (95% CI: 70.7, 112.7%) for clients aged 15–19 years and 83.6% (95% CI: 61.1, 106.2%) for clients aged 20–24 years. Overall, 41.5% of clients had a first PrEP return visit between 21 and 42 days of PrEP initiation.

**Conclusions:**

The high number and proportion of AGYW initiated on PrEP suggests that decentralising PrEP services to DREAMS community centres has the potential to improve PrEP access among AGYW. Increasing HIV risk perception among AGYW may improve PrEP continuation.

## 1 INTRODUCTION

While Zambia has made steady progress towards achieving the UNAIDS 95‐95‐95 goals (89:98:96), an estimated 28,000 new HIV acquisitions still occur annually [1]. Despite accounting for only 20% of Zambia's population, adolescents and young people (AYP) aged 15–24 years account for 40% of all new HIV acquisitions annually, with new HIV acquisitions among females being six times higher than their male counterparts [2]. Multiple factors predispose AYP to HIV acquisition risk, including low comprehensive HIV knowledge, multiple sexual partnerships and age‐mixing sexual relationships, low condom use, sexual intercourse under the influence of alcohol [3], as well as early sexual debut, transactional sex and intimate partner violence (IPV) [3, 4, 5]. Other barriers include poor attitude of healthcare providers which affects access to quality and youth‐friendly sexual and reproductive health services [2, 6].

In response to the increasing incidence of HIV among adolescent girls and young women (AGYW), in 2014, the United States President's Emergency Plan for AIDS Relief (PEPFAR) and partners launched the Determined, Resilient, Empowered, AIDS‐free, Mentored and Safe (DREAMS) initiative to comprehensively address HIV vulnerability among AGYW in communities with the highest HIV prevalence across 10 countries in sub‐Saharan Africa, including Zambia [7, 8]. The DREAMS initiative delivers multi‐layered and linked social asset, biomedical and structural interventions, all aimed at reducing the risk of HIV acquisition among AGYW [7, 9, 10, 11].

In 2016, Zambia adopted pre‐exposure prophylaxis (PrEP)—oral once‐daily tenofovir disoproxil fumarate plus emtricitabine—as part of combination HIV prevention in line with World Health Organization (WHO) guidance [12, 13, 14], and started providing PrEP services in health facilities in 2018. By the end of 2022, 131,493 individuals had initiated PrEP, up from 419 in 2019, with DREAMS contributing 78% of AGYW PrEP initiations [15]. In Zambia, PrEP eligibility is based on the following criteria: not living with HIV but at perceived risk of HIV acquisition arising from vaginal/anal intercourse without condoms; sexually active with a partner known to be living with HIV or at substantial risk of HIV acquisition; sexually active with a partner living with HIV who is not on effective treatment; history of sexually transmitted infection (STI); history of post‐exposure prophylaxis use; and sharing injection material or equipment [14].

Despite the availability of PrEP free of cost in government health facilities in Zambia, PrEP access remains limited, in part due to PrEP availability being largely restricted to antiretrovial viral therapy (ART) service delivery points. More so, PrEP continuation has been a lingering challenge, with continuation reported as low as 25% at 6 months in the general population in 2019 [16], and as low as 47% among key populations (KPs) in 2023 [17].

Differentiated service delivery (DSD) models offer promising avenues for tailoring HIV prevention to the unique needs and preferences of AGYW and for improving PrEP continuation in this priority population. DSD is an individual‐centred approach that moves away from a one‐size‐fits‐all model for providing services to one that is tailored to the unique needs of intended beneficiaries [18]. The DREAMS initiative offers differentiated HIV prevention services characterised by the provision of PrEP in DREAMS centres—non‐clinical, community‐based health promotion centres that provide a range of multi‐layered social asset building, HIV prevention, psychosocial and economic empowerment services. PrEP is provided during 3‐monthly visits to the DREAMS centres with peer‐led support for PrEP continuation. DREAMS initiative models in different countries have been associated with increased PrEP access [19, 20, 21]. In Zimbabwe, a non‐randomised “plausibility” evaluation found AGYW in DREAMS sites were more likely to report ever and ongoing PrEP use, consistent condom use, fewer sexual partners and less IPV [20]. In Zambia, utilisation of DREAMS centres resulted in 77% of AGYW at substantial risk of HIV initiating PrEP, with 68% of them receiving at least one refill, although both PrEP initiation and continuation differed by age group and location [21].

In this paper, we report PrEP uptake and continuation among AGYW receiving a community‐based DREAMS DSD model in seven districts of Zambia through the USAID/PEPFAR‐funded Controlling the HIV Epidemic for Key and Underserved Populations (CHEKUP) I programme. We also examine behavioural, socio‐demographic and programmatic factors associated with PrEP continuation to inform future differentiated PrEP service delivery in Zambia and the region.

## 2 METHODS

### 2.1 USAID CHEKUP I DREAMS programme components

In 2021, the Centre for Infectious Disease Research in Zambia (CIDRZ) started implementing CHEKUP I in partnership with the Young Women Christian Association (YWCA), Copper Rose Zambia, Pact Incorporated, and Tackle Africa. The goal of CHEKUP I is to prevent new HIV acquisition among key and priority populations at risk of HIV, including AGYW. Nested within CHEKUP I was the DREAMS initiative, implemented in 37 communities (i.e. 37 DREAMS centres) in seven districts of Zambia—Livingstone, Lusaka, Ndola, Kitwe, Luanshya, Mufulira and Chingola. These districts were selected by PEPFAR/USAID Zambia because they are all urban districts, lie along the main transport corridor, and were located in provinces with comparatively high HIV prevalence [1].

**Figure 1:**
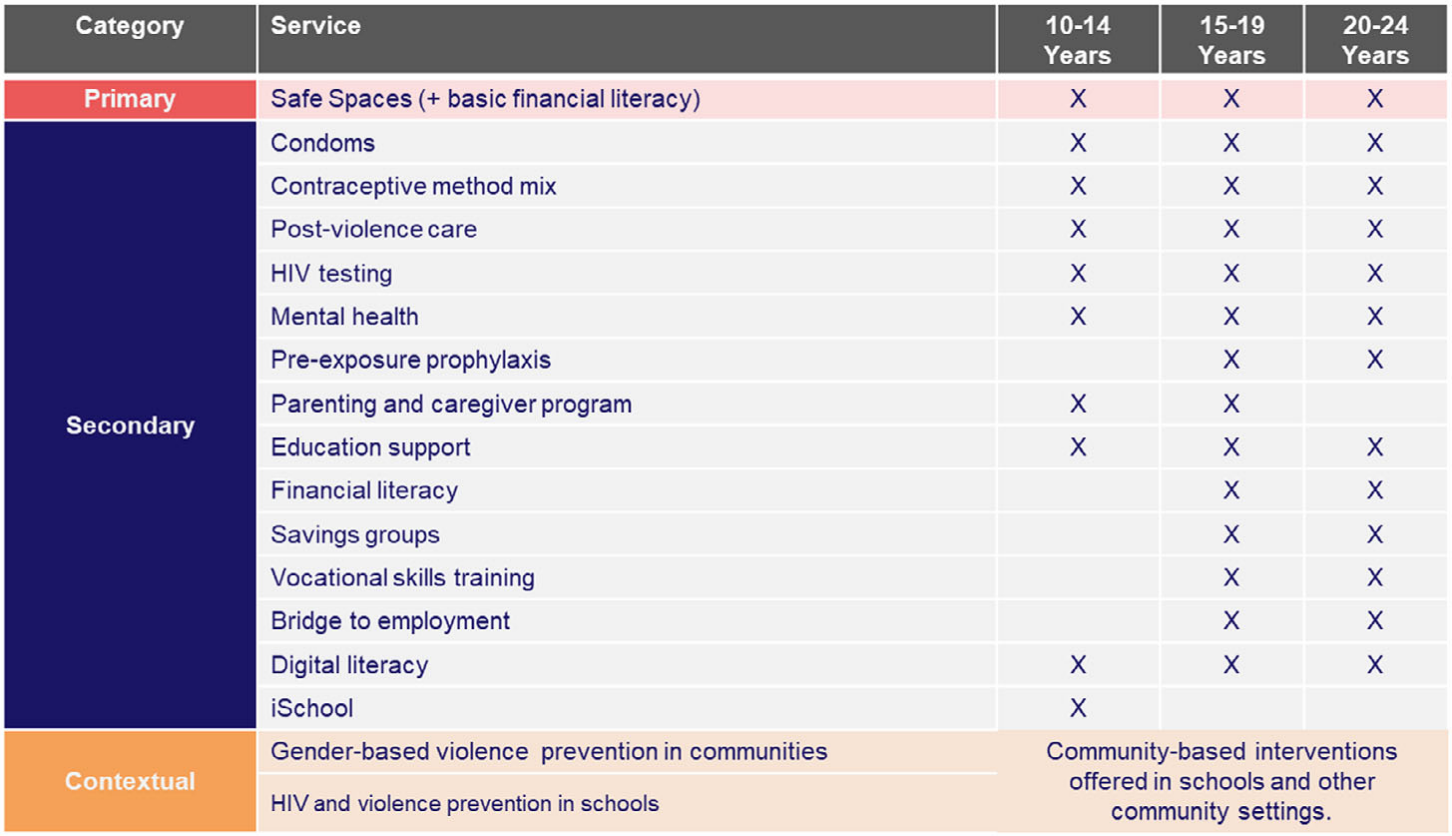
DREAMS Zambia package of core services by age group (reference: USAID CHEKUP I Implementation Plan). Abbreviations: CHEKUP, controlling the HIV epidemic for key and underserved populations; DREAMS, determined resilient, empowered, AIDS‐free, mentored and safe.

The DREAMS initiative offered multiple differentiated interventions to AGYW aged 10–24 years to mitigate the risk of HIV acquisition (Figure 1). We adapted the 13‐session behavioural change Stepping Stones programme as the primary and compulsory package for all AGYW to help promote sexual health, improve psychological wellbeing and prevent HIV acquisition [22]. The Stepping Stones programme has been widely adapted and recommended for use in different settings to address harmful gender norms, gender inequality and harmful interpersonal power dynamics [4]. Depending on their age group, risk profile and interest, the AGYW also received additional (secondary) services such as HIV testing, vocational skills training, digital literacy and educational support (Figure 1). Eligible HIV‐negative AGYW were screened by DREAMS centre nurses for initiation on PrEP. Once initiated on PrEP, DREAMS centre nurses periodically contacted AGYW by phone and occasionally conducted home visits to remind them of upcoming PrEP appointments. Other services provided to AGYW regardless of HIV status were educational support (payment of school fees and uniforms); payment of fees for vocational skills training; digital skills training as a bridge to self or formal employment; linkage to employment opportunities; financial literacy and formation of savings groups; post‐sexual and gender‐based violence care and mental health screening and counselling.

Eligibility for enrolment in DREAMS was based on the vulnerability and risk profile of the AGYW. DREAMS enrolment criteria for 10‐ to 14‐year‐old adolescents were based on any of the following: ever being pregnant, currently pregnant or has a child; being a victim of emotional or physical violence or abuse or neglect; alcohol or drug use; out of school; being an orphan; and engages in sexual activity. For 15‐ to 19‐year‐old AGYW, DREAMS enrolment was based on any of the following criteria: had multiple sexual partners in the past year; ever been pregnant, currently pregnant or has a child; ever been diagnosed with an STI; no or irregular condom use; transactional sex including staying in a relationship for material or financial support; experience of sexual violence (lifetime); engaged in alcohol or drug abuse; out of school; and orphanhood. DREAMS enrolment criteria for 20‐ to 24‐year‐olds were the same as 15‐ to 19‐year‐olds but did not include school status, orphanhood, or current or former pregnancy as criteria.

### 2.2 Innovation: from health facilities to community‐based DREAMS centres—differentiated PrEP service delivery

In August 2022, instead of referring DREAMS beneficiaries to government health facilities for PrEP services, CHEKUP I began to provide PrEP services to AGYW on‐site in community‐based DREAMS centres. The CHEKUP I model of PrEP service delivery followed WHO calls to improve PrEP acceptability, accessibility and continuation [23], and the Zambian Ministry of Health (MOH) DSD models [2, 24]. The CHEKUP I DREAMS programme differentiated approach to PrEP delivery incorporated the following DSD building blocks (Table 1): (1) *When*—monthly after initiation followed by 3 monthly PrEP visits; (2) *Where*—community‐based DREAMS centres to ensure decentralised and expanded access; (3) *Who*—trained young nurses who work full‐time in the DREAMS centres and were supported by peers to facilitate provision of respectful, non‐stigmatising PrEP services; and (4) *What*—PrEP initiation and refills for at‐risk, HIV‐negative AGYW, HIV status monitoring, comprehensive economic strengthening package and other multi‐layered psychosocial support services (Figure 1). Each DREAMS centre was linked to a government health facility, which served as a bridge for HIV treatment and a source of HIV test kits and PrEP medication.

**Table 1:** CHEKUP I PrEP differentiated service delivery building blocks for PrEP continuation

WHEN	Three‐monthly
WHERE	Community‐based Determined Resilient Empowered AIDS‐free, Mentored Safe centres
WHO	Trained young nurses Adolescent girls and young women peers
WHAT	Pre‐exposure prophylaxis refills HIV testing Comprehensive economic strengthening package Multi‐layered psychosocial support services

Abbreviations: CHEKUP, controlling the HIV epidemic for key and underserved populations; PrEP, pre‐exposure prophylaxis.

### 2.3 Data collection and outcomes

Data on PrEP initiation and continuation were collected from programmatic data sources and reported according to PEPFAR indicators for monitoring, evaluation and reporting [25]. The indicators included variables for HIV testing and HIV test results and described each step of the PrEP cascade. These variables captured the number of AGYW enrolled in DREAMS, and the number and proportion of AGYW who: tested HIV negative; were screened for PrEP; found eligible for PrEP; newly initiated PrEP; and returned for PrEP follow‐up or re‐initiation. Data to report against these indicators were collected at individual‐level using a bespoke DHIS2‐compatible data entry system created by CIDRZ and using a unique identifier code that allowed longitudinal client follow‐up across DREAMS centres along the prevention‐to‐care continuum and prevented record duplication. Potential recipients of PrEP services completed a screening form developed in partnership with USAID and PEPFAR, including nine DREAMS enrolment criteria (effectively risk factors) for AGYW aged 15–19 years and six items for AGYW aged 20–24 years (File S1). These DREAMS enrolment criteria formed the basis for the risk analysis presented herein. For this paper, we described PrEP initiation and PrEP continuation, with PrEP initiation defined as documented first‐time PrEP collection at a community‐based DREAMS centre and PrEP continuation defined as documented evidence of ≥ 1 PrEP refill within 180 days of PrEP initiation. Individuals for which age and/or PrEP dispensation dates were not available in the routine record were excluded from the analysis.

### 2.4 Statistical analysis

We analysed de‐identified line‐listed PrEP data for AGYW aged 15–24 years enrolled in DREAMS between 11 August 2022 and 23 August 2024. We defined the date of introduction of our programme innovation as 1 August 2022 when CHEKUP I started providing PrEP services on site within the DREAMS centres. We described beneficiary characteristics and DREAMS enrolment criteria using descriptive statistics. We used Pearson's Chi‐squared test to examine differences in beneficiary characteristics between clients with only a PrEP initiation visit and those with ≥ 2 PrEP visits (i.e. an initiation plus ≥ 1 return visit). We used mixed effects Poisson regression modelling to estimate the association between DREAMS enrolment criteria and PrEP continuation, as well as the association between components of the Zambia DREAMS programme and PrEP continuation adjusting for age, marital status and family planning services accessed (per directed acyclic graph, Figure S1) allowing for random effects at the district level. Using Poisson regression models, we developed marginal probability plots of PrEP continuation by number of DREAM enrolment criteria. Finally, we used Kaplan‐Meier methods to estimate the time to the first PrEP return visit by client age band. All analyses were performed using Stata (StataCorp LLC, College Station, TX, USA, release 18).

### 2.5 Ethical considerations

The paper used de‐identified data routinely available through the CHEKUP I programme. Ethical approval for this study was granted by the ERES Converge IRB (#2023‐Oct‐009) and the National Health Research Authority of the Ministry of Health (NHRA0003/19/10/23), without requiring patient consent given the use of de‐identified, routinely collected data.

## 3 RESULTS

### 3.1 DREAMS enrolment and PrEP eligibility

**Figure 2:**
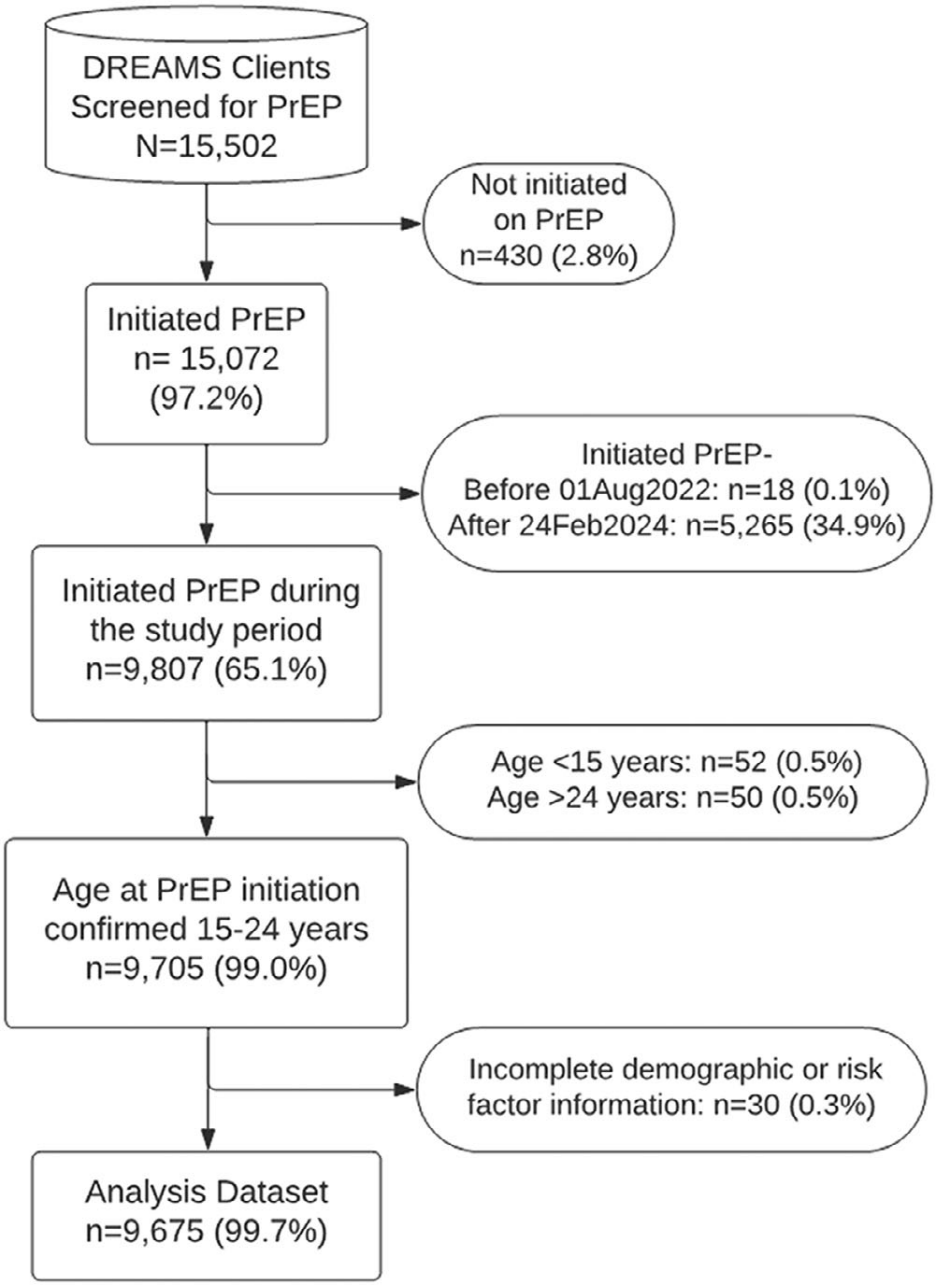
Flow chart of Determined Resilient, Empowered, AIDS‐free, Mentored and Safe (DREAMS) adolescent girls and young women (AGYW) beneficiaries of differentiated pre‐exposure prophylaxis (PrEP) services.

Between 11 August 2022 and 23 August 2024, we screened 15,502 AGYW for PrEP eligibility based on their HIV risk profile ascertained during DREAMS programme screening and enrolment. Out of these, 15,072 (97.2%) were initiated on PrEP per MOH PrEP guidelines, and 430 (2.8%) were not initiated on PrEP (Figure 2). Of those initiated on PrEP, 9807 (65.1%) had sufficient follow‐up time to allow for observation of a PrEP return visit within 180 days of initiation. We excluded 5265 (34.9%) AGYW who initiated PrEP after 23 February 2024 and did not contribute sufficient follow‐up time and 18 (0.1%) who had initiated PrEP before the introduction of our intervention. Of those initiated on PrEP, 9705 (99.0%) were confirmed 15–24 years of age at PrEP initiation, and 9675 (99.7%) had complete data for primary analyses.

### 3.2 Baseline characteristics of DREAMS clients who initiated PrEP

Of the 9675 AGYW with complete data analysed, the median age was 21 years (interquartile range: 20–23), with 5.7% under the age of 18 at initiation (Table 2). The majority (83.2%) of PrEP initiators were between 20 and 24 years old; 81.7% were single; and half of AGYW were initiated on PrEP in 2024, showing an upward trend of PrEP initiations following the introduction of our PrEP DSD model. Almost one‐third (32.0%) of AGYW initiated on PrEP were from Lusaka district, which, as the country's capital and largest urban centre in Zambia, was expected, followed by Ndola district, at 17.9%, the third largest city in Zambia (Table 2).

**Table 2:** DREAMS beneficiary characteristics by PrEP continuation status, August 2022–August 2024 (*N* = 9675)

*Category*	*Factor*	*Level*	*No return visit/refill within 180 days of initiation*	*≥ 1 PrEP return visit/refill within 180 days of initiation*	*Total*
			*n (%)*	*n (%)*	*N (%)*
			*n* = 3969	*n* = 5706	*N* = 9675
Demographic characteristics	Enrolment age	Median (IQR)	21 (20–23)	21 (20–23)	21 (20–23)
	Enrolment age (categorically)	15–19 years	775 (19.5)	846 (14.8)	1621 (16.8)
		20–24 years	3194 (80.5)	4860 (85.2)	8054 (83.2)
	Marital status	Married	675 (17.0)	1063 (18.6)	1738 (18.0)
		Single	3277 (82.6)	4625 (81.1)	7902 (81.7)
		Separated/divorced	16 (0.4)	16 (0.3)	32 (0.3)
		Widowed	1 (0.0)	2 (0.0)	3 (0.0)
Other service details	Accessed family planning services	No	2539 (64.0)	3380 (59.2)	5919 (61.2)
		Yes	1430 (36.0)	2326 (40.8)	3756 (38.8)
	DREAMS district	Chingola District	534 (13.5)	431 (7.6)	965 (10.0)
		Kitwe District	640 (16.1)	835 (14.6)	1475 (15.2)
		Livingstone District	346 (8.7)	679 (11.9)	1025 (10.6)
		Luanshya District	151 (3.8)	459 (8.0)	610 (6.3)
		Lusaka District	1479 (37.3)	1615 (28.3)	3094 (32.0)
		Mufulira District	231 (5.8)	542 (9.5)	773 (8.0)
		Ndola District	588 (14.8)	1145 (20.1)	1733 (17.9)
Wrap‐around services	Received financial literacy module	No	3354 (84.5)	4708 (82.5)	8062 (83.3)
		Yes	615 (15.5)	998 (17.5)	1613 (16.7)
	Received saving literacy module	No	3643 (91.8)	5357 (93.9)	9000 (93.0)
		Yes	303 (7.6)	323 (5.7)	626 (6.5)
		Missing	23 (0.6)	26 (0.5)	49 (0.5)
	Received digital literacy module	No	3807 (95.9)	5450 (95.5)	9257 (95.7)
		Yes	162 (4.1)	256 (4.5)	418 (4.3)
DREAMS enrolment responses/risk factors	Multiple sexual partners in the past 12 months (screen)	No	3197 (82.0)	4734 (81.9)	7931 (82.0)
		Yes	701 (18.0)	1043 (18.1)	1744 (18.0)
	Current or past pregnancy or has child (screen)	No	737 (18.9)	805 (13.9)	1542 (15.9)
		Yes	28 (0.7)	45 (0.8)	73 (0.8)
		Missing	3133 (80.4)	4927 (85.3)	8060 (83.3)
	Ever diagnosed with STI (screen)	No	3784 (97.1)	5570 (96.4)	9354 (96.7)
		Yes	114 (2.9)	207 (3.6)	321 (3.3)
	No or irregular condom use (screen)	No	1183 (30.3)	1693 (29.3)	2876 (29.7)
		Yes	2715 (69.7)	4084 (70.7)	6799 (70.3)
	Receive material or financial support for sex (screen)	No	3472 (89.1)	5077 (87.9)	8549 (88.4)
		Yes	426 (10.9)	700 (12.1)	1126 (11.6)
	Experience of sexual violence (screen)	No	3696 (94.8)	5400 (93.5)	9096 (94.0)
		Yes	202 (5.2)	377 (6.5)	579 (6.0)
	Alcohol or drug abuse (screen)	No	3007 (77.1)	4232 (73.3)	7239 (74.8)
		Yes	891 (22.9)	1545 (26.7)	2436 (25.2)
	Out of school (screen)	No	747 (19.2)	804 (13.9)	1551 (16.0)
		Yes	17 (0.4)	47 (0.8)	64 (0.7)
		Missing	3134 (80.4)	4926 (85.3)	8060 (83.3)
	Orphanhood (screen)	No	648 (16.6)	752 (13.0)	1400 (14.5)
		Yes	117 (3.0)	100 (1.7)	217 (2.2)
		Missing	3133 (80.4)	4925 (85.3)	8058 (83.3)
	Count of DREAMS enrolment/risk factors	1	2736 (69.5)	3685 (65.1)	6421 (66.4)
		2	1072 (27.2)	1676 (29.6)	2748 (28.4)
		3	119 (3.0)	257 (4.5)	376 (3.8)
		4	7 (0.2)	36 (0.6)	43 (0.4)
		5	2 (0.1)	6 (0.1)	8 (0.1)
		6	0 (0.0)	2 (0.0)	2 (0.0)
		Unknown	30 (0.8)	43 (0.8)	73 (0.1)

Abbreviations: DREAMS, Determined Resilient, Empowered, AIDS‐free, Mentored and Safe; IQR, interquartile range; PrEP, pre‐exposure prophylaxis; STI, sexually transmitted infection.

### 3.3 Risk profile of AGYW initiated on PrEP

Around 18% of PrEP initiators reported multiple sexual partnerships in the past 12 months; two‐thirds (70.3%) reported no or irregular condom use; one‐quarter (25.2%) reported unhealthy alcohol or drug use; slightly over 11.6% reported engaging in transactional sex; and 6.0% reported lifetime experience of sexual violence (Table 2).

### 3.4 PrEP continuation and associated factors

The proportion of AGYW who had ≥ 1 return visit for a PrEP refill within 180 days of initiation was 59.0% (*n*/*N* = 5706/9675). After adjusting for age, marital status and family planning service access, clients 20–24 years old were 1.23 (95% CI: 1.07, 1.42) times as likely to return for a PrEP refill during the specified time frame than beneficiaries 15–19 years old. Notably, DREAMS programme “wrap‐around” services such as economic strengthening were not statistically significantly associated with return PrEP visit. Though not statistically significant, receipt of saving literacy training was associated with a lower likelihood (aPR: 0.74, 95% CI: 0.23, 1.91) of a return PrEP visit (Table 3).

**Table 3:** Regression for at least one return PrEP visit within 180 days of initiation (*N* = 9675)

*Factor*	*Level*	Unadjusted			Adjusted		
		*PR*	*95% CI*	*p‐value*	*aPR*	*95% CI*	*p‐value*
Age	15–19 years	1.00 (ref)	–	–	1.00 (ref)	–	–
	20–24 years	1.23	(1.08, 1.41)	0.003	1.23	(1.07, 1.42)	0.005
Marital status	Married	1.00 (ref)	–	–	1.00 (ref)	–	–
	Single	0.97	(0.94, 1.01)	0.109	1.00	(0.96, 1.05)	0.948
	Separated/divorced	0.85	(0.63, 1.15)	0.303	0.86	(0.63, 1.18)	0.358
	Widowed	0.99	(0.46, 2.14)	0.986	0.97	(0.46, 2.05)	0.941
Accessed family planning services	No	1.00 (ref)	–	–	1.00 (ref)	–	–
	Yes	1.05	(0.97, 1.13)	0.215	1.03	(0.96, 1.12)	0.399
Received financial literacy module	No	1.00 (ref)	–	–	1.00 (ref)	–	–
	Yes	1.03	(0.95, 1.12)	0.476	1.02	(0.32, 1.75)	0.645
Received saving literacy module	No	1.00 (ref)	–	–	1.00 (ref)	–	–
	Yes	0.77	(0.33, 1.77)	0.535	0.74	(0.23, 1.91)	0.499
Received digital literacy module	No	1.00 (ref)	–	–			
	Yes	0.92	(0.66, 1.28)	0.617	0.92	(0.66, 1.28)	0.628

*Note*: Adjustment set—age, marital status, family planning access and district of DREAMS community centre (as random effect).

Abbreviations: aPR, adjusted prevalence ratio; CI, confidence interval; DREAMS, Determined Resilient, Empowered, AIDS‐free, Mentored and Safe; PR, prevalence ratio; STI, sexually transmitted infection.

DREAMS clients who reported being out of school were 1.35 (95% CI: 1.20, 1.52) times as likely to have a return PrEP visit compared to those who reported being in school (Table 4). Though not statistically significant, clients self‐identifying as being an orphan were less likely (PR: 0.81, 95% CI: 0.66, 1.00) to have a return PrEP visit compared to counterparts who did not self‐identify as such (Table 4).

**Table 4:** Regression results for PrEP continuation (defined as follow‐up within 180 days of next appointment) by screening factor for DREAMS enrolment (*N* = 9675)

*Factor*	*Level*	*PR*	*95% CI*	*p‐value*
Multiple sex partners last 12 months	No	1.00 (ref)	–	–
	Yes	1.02	(0.93, 1.11)	0.680
Ever pregnant^a^	No	1.00 (ref)	–	–
	Yes	1.16	(0.82, 1.63)	0.394
STI diagnosis	No	1.00 (ref)	–	–
	Yes	1.03	(0.92, 1.16)	0.739
Inconsistent/no condom use	No	1.00 (ref)	–	–
	Yes	1.02	(0.97, 1.08)	0.098
Transactional sex	No	1.00 (ref)	–	–
	Yes	1.02	(0.93, 1.12)	0.919
Experienced sexual violence	No	1.00 (ref)	–	–
	Yes	1.06	(1.00, 1.14)	0.058
Alcohol and/or drug abuse	No	1.00 (ref)	–	–
	Yes	1.05	(0.99, 1.13)	0.182
Out of school^a^	No	1.00 (ref)	–	–
	Yes	1.35	(1.20, 1.52)	< 0.001
Orphanhood^a^	No	1.00 (ref)	–	–
	Yes	0.81	(0.66, 1.00)	0.051

Abbreviations: CI, confidence interval; PrEP, pre‐exposure prophylaxis; PR, prevalence ratio; STI, sexually transmitted infection.

^a^ Only asked of population aged 15–19 years (*N* = 1617).

**Figure 3:**
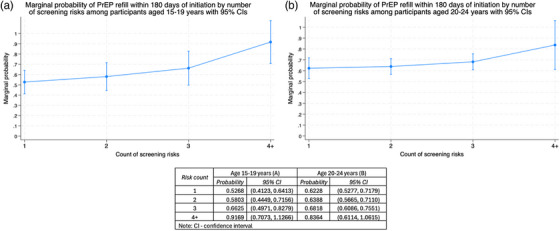
**(a)** Marginal probability of pre‐exposure prophylaxis (PrEP) continuation by risk factor among clients aged 15–19 years. **(b)** Marginal probability of PrEP continuation by risk factor among clients aged 20–24 years (Table S1). Point probability estimates and associated 95% confidence intervals by age category.

Across age bands, the percent probability of having a PrEP return visit within 180 days of initiation was highest among clients who reported ≥ 4 DREAMS enrolment criteria/risk factors at 91.7% (95% CI: 70.7, 112.7%) for clients aged 15–19 years and 83.6% (95% CI: 61.1, 106.2%) for clients aged 20–24 years (Figure 3). Probability of return PrEP visit varied significantly by the number of DREAMS enrolment/risk factors among clients in the 15–19 years age band. Specifically, the percent probability of a PrEP return visit among individuals with ≥ 4 DREAM enrolment criteria/risk factors was 91.7% (95% CI: 70.7, 112.7%), higher than among clients with one risk factor (52.7%, 95% CI: 41.2, 64.1%) (Figure 3). Thus, the lower the number of DREAM enrolment criteria/risk factors reported, the lower the marginal probability of a PrEP follow‐up visit, among clients aged 15–19 years.

**Figure 4:**
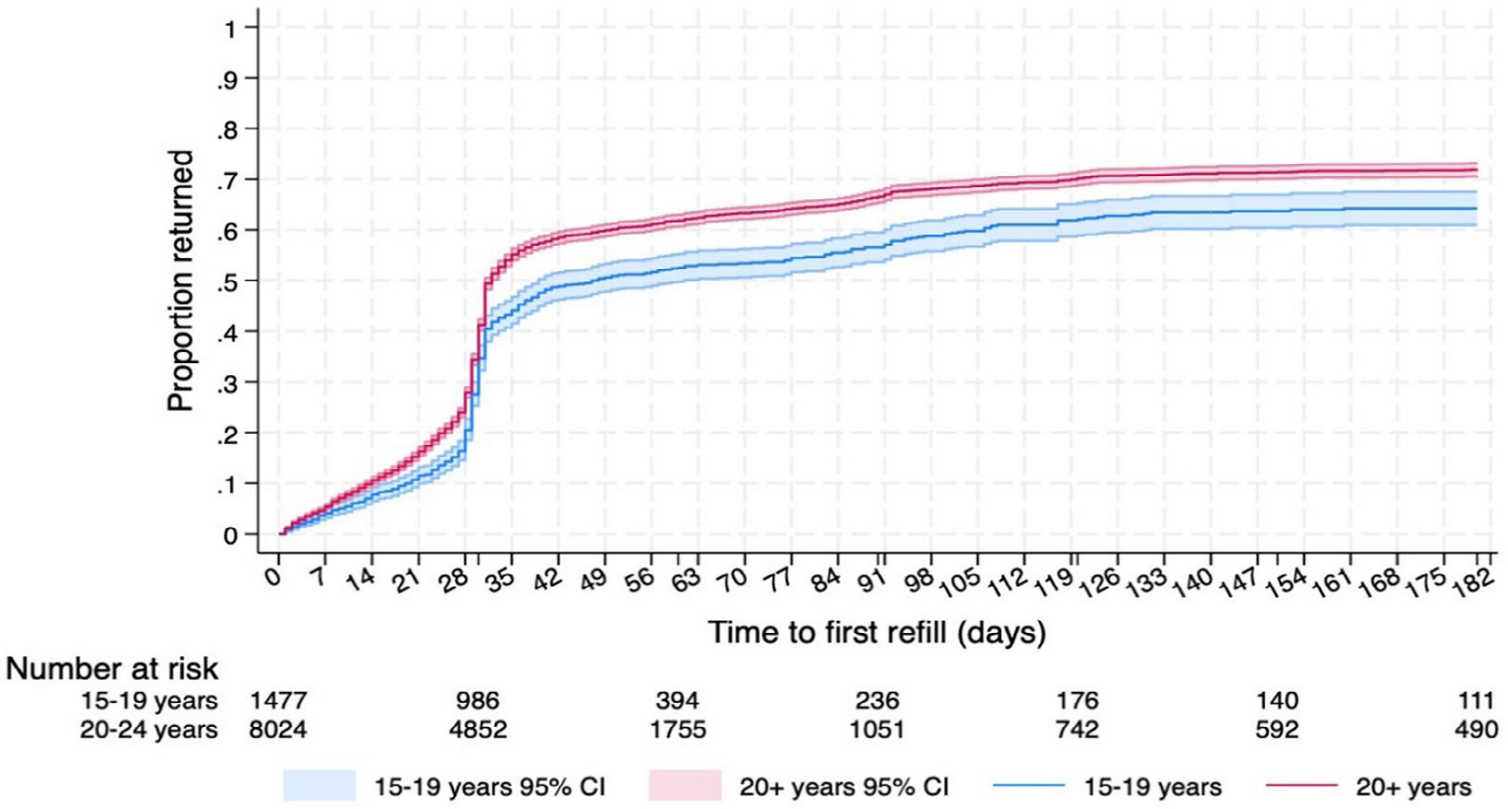
Kaplan‐Meier estimates for time to first pre‐exposure prophylaxis (PrEP) return visit with accompanying 95% confidence intervals (CI) by age band.

Older AGYW had an overall higher proportion of return for the first return PrEP visit within the first 180 days after PrEP initiation compared to AGYW in the younger age band (Figure 4). Overall, 41.5% of the clients had a first return PrEP visit between 21 and 42 days after PrEP initiation. A total of 13.7% of clients had a first return PrEP visit between 42 and 180 days after initiation.

## 4 DISCUSSION

In this report, we describe DREAMS centres as a community‐based model for differentiated PrEP service delivery among AGYW in Zambia. We note the high numbers of AGYW initiating and continuing PrEP in CHEKUP I DREAMS programme, with PrEP initiation reaching nearly 10,000 AGYW in 37 communities in seven districts of Zambia. Differentiated PrEP delivery relied upon DSD building blocks involving full‐time young nurses supported by AGYW peers who provided HIV testing and PrEP refills to clients at DREAMS centres located in the community. We observed higher PrEP continuation among AGYW who reported a higher number of DREAMS enrolment criteria, which are risk factors for HIV acquisition, and might suggest alignment of PrEP use with self‐perceptions of HIV risk.

The high number of AGYW initiating PrEP in our DREAMS programme corroborates evidence from similar DSD programmes in Zambia [15, 17, 21] and the region [26, 27, 28, 29, 30]. In Zambia, by the end of 2022, 78% of all AGYW on PrEP had received PrEP through a DREAMS centre [15]. The high PrEP initiation rate observed in our programme suggests a large unmet need for PrEP among AGYW, and the willingness of AGYW to adopt this service when it is made easily accessible and differentiated to their unique needs and preferences as opposed to lower PrEP uptake when PrEP offerings are limited to traditional health facilities providing ART services [15]. Our finding of community‐based DREAMS centres supporting the uptake of differentiated PrEP services for AGYW aligns with results from a similar community‐based intervention in Zambia in which the number of KP accessing PrEP markedly increased following the introduction of venue‐based PrEP services [17]. Taken together, these findings underscore the utility of differentiated approaches for expanding access to community‐based PrEP services in Zambia.

Notwithstanding the high PrEP uptake at the first visit in our DREAMS programme, PrEP continuation decreased over time. We conjecture that this decrease in PrEP continuation could have been due to not all AGYW remaining at risk and needing PrEP. Our analysis found that the availability of other DREAMS support services such as social asset building and economic strengthening interventions were not significantly associated with the likelihood of a return PrEP visit. Instead, PrEP continuation varied by cumulative self‐reported HIV acquisition risk and social vulnerability. Across all age bands (15–19 and 20–24 years), AGYW who reported multiple risk DREAMS enrolment criteria/risk factors were more likely to continue PrEP compared to those who reported fewer such risk factors. Similarly, older AGYW (20–24 years) had an overall higher proportion of return for the PrEP in the first 180 days after PrEP initiation compared to younger AGYW (15–19 years).

The relatively low number of AGYW continuing on PrEP fits with other evidence from Zambia [21] and other African regions [30, 31]. Factors as varied as medication attributes and pill burden [31], stigma and discrimination [32, 33], and concerns about people conflating PrEP with ART [34] negatively affecting PrEP continuation. Therefore, improving PrEP continuation requires recognising the heterogeneity among AGYW in terms of their socio‐demographics, HIV risk perceptions, experiences and tailoring service delivery to their unique circumstances. The introduction of new injectable PrEP agents, such as cabotegravir [35] and lenacapavir [36], may offer a more acceptable, anonymous and longer‐acting formulation to assuage stigma, concerns about pill burden and challenges of disclosure.

Our study was not without limitations. First, due to periodic PrEP stock‐outs, the programme had to ration the provision of PrEP services. This could have undermined beneficiary interest to return for PrEP and/or could have created concerns about long‐term PrEP availability, either of which could have influenced PrEP initiation and continuation. Second, we conceptualised DREAMS enrolment criteria to be equally additive risk factors in our regression analysis, with limited empiric evidence to support this assumption, although this type of risk appraisal is operationally useful in DREAMS programme settings where simple risk screening tools are used to help providers decide on programme eligibility. Third, while a screening and enrolment tool was administered to characterise AGYW's risk profiles, the social desirability not to be viewed as sexually active could have resulted in the under‐reporting of the enrolment criteria we examined in the study. Fourth, this is a descriptive study that did not include a comparator group. Therefore, the effect of our differentiated, DREAMS‐based PrEP model compared to standard health facility‐based PrEP services could not be ascertained. Despite these limitations, our report adds to the body of knowledge on the utility of community‐based DSD models to promote equity of access to HIV prevention services.

As countries scale‐up PrEP, further research is needed to assess the effectiveness of various differentiated PrEP delivery models to inform HIV prevention programming and policy. Questions remain about the implementation strategies needed to increase the likelihood of PrEP continuation, such as the optimal frequency, cadence and delivery modality (e.g. home delivery, mobile, DREAMS centre, etc.) for providing PrEP, the impact of support groups and peer navigation on PrEP adherence and continuation, and the effects of “wrap‐around” services on PrEP outcomes. These questions must be answered to optimally differentiate PrEP services to reduce stubbornly high HIV incidence among AGYW.

## 5 CONCLUSIONS

We have described community DREAMS centres as a differentiated model to improve access to PrEP services for AGYW in Zambia. We observed relatively high rates of PrEP initiation, but a need to improve PrEP continuation. Our analysis has also shown an association between DREAMS enrolment criteria at baseline and PrEP continuation, suggesting that the degree of HIV risk and/or risk perception may be aligned with PrEP use among AGYW in our programme. As countries scale‐up PrEP, differentiating prevention services will be crucial to improving access for populations that remain marginalised and underserved.

## COMPETING INTERESTS

None of the authors have any competing interests.

## AUTHORS’ CONTRIBUTIONS

MM led the CHEKUP I DREAMS programme management, conceptualised and led the writing of the manuscript. All authors contributed to PrEP service delivery as part of the implementing team. MM led the writing and coordination with support from MEH. WJM and KC were responsible for data extraction cleaning. JMP led the data analysis with support from WJM and KC. All authors reviewed and contributed to the writing.

## FUNDING

The CHEKUP I project and this publication have been supported by the U.S. President's Emergency Plan for AIDS Relief (PEPFAR) through the United States Agency for International Development (USAID) under the terms of Cooperative Agreement 72061121CA00002.

## DISCLAIMER

The findings and conclusions of this paper are those of the authors and do not necessarily represent the official position of the funding agency.

## Supporting information




**Table S1**: Marginal probability estimates and 95% confidence intervals.
**Figure S1**: Directed acyclic graph (DAG) for relationship between wrap‐around services and pre‐exposure prophylaxis refill/persistence.

## Data Availability

The data that support the findings of this study are available on request from the corresponding author. The data are not publicly available due to privacy or ethical restrictions.
