# Supplementary material for: Improving PrEP access for adolescent girls and young women: a descriptive analysis of community‐based PrEP delivery in the DREAMS programme in Zambia
Source: J Int AIDS Soc. 2025 Jul 7;28(Suppl 3):e26484. doi: 10.1002/jia2.26484 (PMC12232481; doi:10.1002/jia2.26484)
Supplement: Supplementary file 1 — Table S1: Marginal probability estimates and 95% confidence intervals. Figure S1: Directed acyclic graph (DAG) for relationship between wrap‐around services and pre‐exposure prophylaxis refill/persistence. [file JIA2-28-e26484-s001.docx]

**Supporting Information**

**Table S1.** Marginal probability estimates and 95% confidence intervals

| *Risk count* | Age 15-19 years | | Age 20-24 years | |
| --- | --- | --- | --- | --- |
|  | *Probability* | *95% CI* | *Probability* | *95% CI* |
| 1 | 0.5268 | (0.4123, 0.6413) | 0.6228 | (0.5277, 0.7179) |
| 2 | 0.5803 | (0.4449, 0.7156) | 0.6388 | (0.5665, 0.7110) |
| 3 | 0.6625 | (0.4971, 0.8279) | 0.6818 | (0.6086, 0.7551) |
| 4+ | 0.9169 | (0.7073, 1.1266) | 0.8364 | (0.6114, 1.0615) |
| Note: CI - confidence interval | | | | |

| *Risk count* | Age 15-19 years (A) | | Age 20-24 years (B) | |
| --- | --- | --- | --- | --- |
|  | *Probability* | *95% CI* | *Probability* | *95% CI* |
| 1 | 0.5268 | (0.4123, 0.6413) | 0.6228 | (0.5277, 0.7179) |
| 2 | 0.5803 | (0.4449, 0.7156) | 0.6388 | (0.5665, 0.7110) |
| 3 | 0.6625 | (0.4971, 0.8279) | 0.6818 | (0.6086, 0.7551) |
| 4+ | 0.9169 | (0.7073, 1.1266) | 0.8364 | (0.6114, 1.0615) |
| Note: CI - confidence interval | | | | |

**Figure S1.** Directed acyclic graph (DAG) for relationship between wrap-around services and pre-exposure prophylaxis (PrEP) refill/continuation.


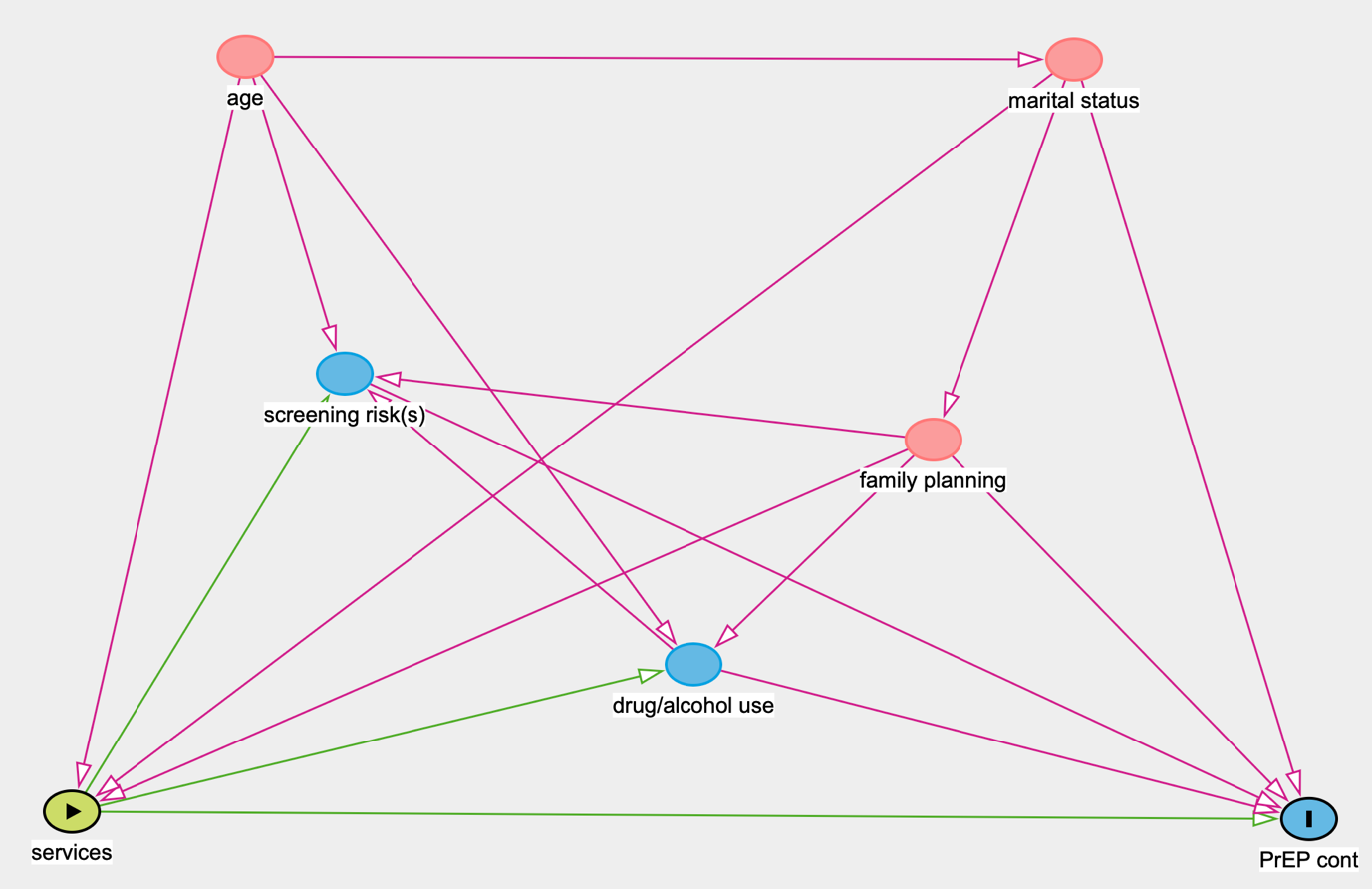


Note: The DAG suggests a minimal adjustment set to evaluate the relationship between the exposure of interest (services provided) and outcome of interest (PrEP continuation) including age, marital status, and family planning uptake.
